# Vocal repertoire and individuality in the plains zebra (*Equus quagga*)

**DOI:** 10.1098/rsos.240477

**Published:** 2024-07-10

**Authors:** Bing Xie, Virgile Daunay, Troels C. Petersen, Elodie F. Briefer

**Affiliations:** ^1^ Behavioural Ecology Group, Section for Ecology and Evolution, University of Copenhagen, Copenhagen, Denmark; ^2^ Research and Conservation, Copenhagen Zoo, Roskildevej 38, 2000 Frederiksberg, Denmark; ^3^ Laboratoire Dynamique du Langage, CNRS, University Lumière Lyon 2, Lyon, France; ^4^ ENES Bioacoustics Research Lab, CRNL, CNRS, Inserm, University of Saint-Etienne, 42100 Saint-Etienne, France; ^5^ Niels Bohr Institute, University of Copenhagen, Copenhagen, Denmark

**Keywords:** vocalization type, vocal individuality, supervised machine learning, unsupervised machine learning, ungulate, bioacoustics

## Abstract

Acoustic signals are vital in animal communication, and quantifying them is fundamental for understanding animal behaviour and ecology. Vocalizations can be classified into acoustically and functionally or contextually distinct categories, but establishing these categories can be challenging. Newly developed methods, such as machine learning, can provide solutions for classification tasks. The plains zebra is known for its loud and specific vocalizations, yet limited knowledge exists on the structure and information content of its vocalzations. In this study, we employed both feature-based and spectrogram-based algorithms, incorporating supervised and unsupervised machine learning methods to enhance robustness in categorizing zebra vocalization types. Additionally, we implemented a permuted discriminant function analysis to examine the individual identity information contained in the identified vocalization types. The findings revealed at least four distinct vocalization types—the ‘snort’, the ‘soft snort’, the ‘squeal’ and the ‘quagga quagga’—with individual differences observed mostly in snorts, and to a lesser extent in squeals. Analyses based on acoustic features outperformed those based on spectrograms, but each excelled in characterizing different vocalization types. We thus recommend the combined use of these two approaches. This study offers valuable insights into plains zebra vocalization, with implications for future comprehensive explorations in animal communication.

## 1. Introduction

Acoustic communication plays an important role in various aspects of animals’ lives, including courtship and mating, offspring care, territorial defence, predator defence, group cohesion, decision making and emotion expression [1–9]. Quantifying and comparing species-specific vocalizations are thus fundamental to understand animal behaviour, communication and ecology [10–13]. Acoustic signals can be classified into different categories based on their acoustic structures and/or corresponding function or context of emission. For example, at the species level, animals share a vocal repertoire consisting of distinct types of vocalizations [14–16]. These distinct vocalizations will often serve specific functions (e.g. contact and mating), and vary in both the amount and category of information that they convey (i.e. their information content), such as static information (stable over time; e.g. individuality [17,18] and sex [19]) and dynamic information (variable over time; e.g. emotion and motivation [20]) about the caller [21].

Vocal repertoires are notoriously difficult to establish, as variations in acoustic signals arise across individuals and environments [22]. Additionally, vocalization types are often graded, and hence may not fall into distinct categories [23]. Newly developed analysis tools provide researchers with improved options for classifying tasks (i.e. acoustic signals) [24]. For example, machine learning offers both supervised and unsupervised tools for classification, where supervised learning categorizes data into predetermined classes, while unsupervised learning recognizes inherent patterns for grouping clusters without prior class labels [25]. Moreover, short-time Fourier transform (STFT), convolutional neural network (CNN) and spectrogram-based unsupervised learning expand applications for acoustic signals, from extracted features to spectrogram, providing opportunities to classify or cluster vocalizations based on the whole structure [22,26–28].

Vocalizations can be particularly important in socially complex species, such as the plains zebra, a species characterized by a complex multi-level social structure [29,30]. Understanding how members of this near-threatened species use vocalizations in this complex system is essential for studying their communication and social dynamics [31]. However, studies on plains zebra acoustic communication are scarce, and the information content of their vocalizations has not been investigated yet. To our knowledge, so far, only two studies have attempted to establish plains zebras’ vocal repertoire, and detected 4–6 distinct vocalization types by subjectively describing vocalizations and contexts of production [29,32]. This study aimed to re-visit the vocal repertoire of plains zebras using modern methods of classifications, and to investigate the individuality content of the resulting vocalization types. Based on previous literature and preliminary field observations, we hypothesized that zebras use at least four distinct vocalization types. We also predicted that the individual distinctiveness would differ between vocalization types, as found in other species (e.g. red-capped mangabeys (*Cercocebus torquatus*) [33], zebra finches (*Taeniopygia guttata*) [34], southern white rhinoceros (*Ceratotherium simum simum*) [35], concave-eared torrent frogs (*Odorrana tormota*) [36], and little auks (*Alle alle*) [37]).

## 2. Method

### 2.1. Data collection and sampling

We collected data in three locations, in Denmark and South Africa: (i) 10 months between December 2020 and July 2021 and between September and December 2021, at Pilanesberg National Park (PNP), South Africa, covering both dry season (i.e. May–September) and wet season (i.e. October–April) [38]; (ii) 16 days between May and June 2019, and 33 days between February and May 2022, at Knuthenborg Safari Park (KSP), Denmark, covering periods both before the park’s opening for tourists (i.e. November–March) and after (i.e. April–October); and (iii) 4 days in August 2019 at Givskud Zoo (GKZ), Denmark.

For all places and periods, three types of data were collected as follows: (i) pictures were taken for each individual from both sides using a camera (Nikon COOLPIX P950); (ii) contexts of vocal production were recorded through either notes (in the first period of KSP and in GKZ) or videos (in the second period of KSP and in PNP) filmed by a video camera recorder (Sony HDRPJ410 HD); (iii) audio recordings were collected using a directional microphone (Sennheiser MKH-70 P48, with a frequency response of 50–20 000 Hz (±2.5 dB)) linked to an audio recorder (Marantz PMD661 MKIII).

Six zebras housed in GKZ were recorded while being separated from one another into three enclosures (the stable, the small enclosure and the savannah) manually by the zookeeper for management purpose, which triggered vocalizations. These vocalizations, along with other types of data, were recorded at distances of 5–30 m.

In KSP, 15–18 zebras (population changed owing to newborns, deaths or removal of adult males) were living with other herbivores in a 0.14 km^2^ savannah. There, we approached the zebras by driving down the road until approximately 7–40 m, at which point spontaneous vocalizations and other information were collected. This distance allowed us to collect good-quality recordings without eliciting any obvious reactions from the zebras to our presence.

Finally, PNP is a 580 km^2^ national park, with approximately 800–2000 zebras [39]. In this park, we drove on the road and parked at distances of 10–80 m when encountering zebras, where all data, including spontaneous vocalizations, were recorded.

### 2.2. Data processing

Individual zebras were manually identified based on the pictures collected from KSP and GKZ (15–18 and 6 zebras, respectively). In PNP, the animals present in the pictures were individually identified using WildMe (https://zebra.wildme.org/), a Web-based machine learning platform facilitating individual recognition. All zebra pictures were uploaded to the platform for a full comparison through the algorithm. The resulting matching candidates were then determined by manually reviewing the output.

**Figure 1.**
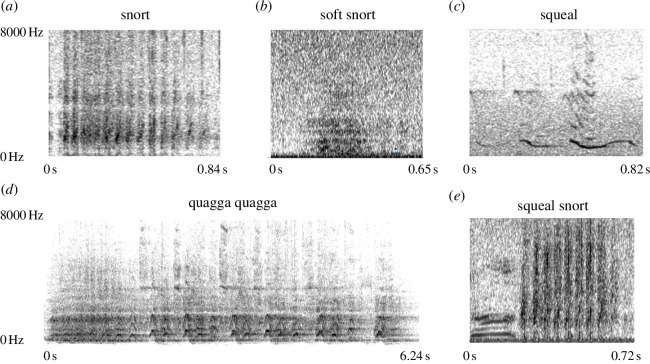
Example spectrograms for each manually labelled vocalization type: (*a*) ‘snort’; (*b*) ‘soft snort’; (*c*) ‘squeal’; (*d*) ‘quagga quagga’; and (*e*) ‘squeal-snort’. Spectrogram settings: number of time steps = 1000, number of frequency steps = 250 and window shape = Gaussian. Corresponding audio files are included in the electronic supplementary material (supplementary audio).

Audio files (sampling rate: 44 100 Hz) were saved at 16-bit amplitude resolution in WAV format. We annotated zebra vocalizations, along with the context of production and individuals emitting the vocalizations, using Audacity software (v. 3.3.3) [40]. Vocalizations were first subjectively labelled as five vocalization types based on both audio and spectrogram examinations (i.e. visual inspection) (table 1 and figure 1). Among these types, the ‘squeal-snort’ was excluded from further analysis, as the focus of this study was on individual vocalization types instead of combinations.

**Table 1.** Subjectively labelled vocalization types.

vocalization type	description	context
snort (figure 1*a*)	a nasal-clearing sound with vibration pulses visible on the spectrogram	appearing in diverse contexts, including grazing, moving, standing and lying
soft snort (figure 1*b*)	a soft exhalation of air resembling white noise on the spectrogram	appearing in similar contexts as the ‘snort’
squeal (figure 1*c*)	a relatively short and high-fundamental-frequency vocalization	mainly emitted during social interactions
quagga quagga (figure 1*d*)	a long series of inhalations and exhalations (‘a-ha’)	mainly uttered during separation
squeal-snort (figure 1*e*)	a temporal combination of a squeal and a snort	appearing in similar contexts as the ‘snort’

### 2.3. Acoustic analysis

We extracted vocalizations of good quality, defined as vocalizations with clear spectrograms, low background noise and no overlap with other sounds, and saved them as distinct audio files. For the individual distinctiveness analysis, we excluded individuals with fewer than five vocalizations of each type, to avoid strong imbalance, resulting in 359 snorts from 28 individuals and 138 squeals from 14 individuals (electronic supplementary material, tables S3 and S4) [35,41]. The individuality content of quagga quagga and soft snorts could not be explored, owing to insufficient individual data. For vocal repertoire analysis, we excluded vocalizations longer than 1.25 s to improve spectrogram-based analysis, following Thomas *et al.* [28]. In total, we gathered 678 vocalizations for the spectrogram-based vocal repertoire analysis, including 117 quagga quagga, 204 snorts, 161 squeals and 196 soft snorts (electronic supplementary material, table S2). Among these vocalizations, six squeals were excluded in the acoustic feature-based vocal repertoire analysis, owing to missing data for one of the features (amplitude modulation extent).

All calls were first high-passed filtered above 30 Hz for snorts and soft snorts, above 500 Hz for squeals and above 600 Hz for quagga quagga (i.e. above the average minimum fundamental frequency of these vocalizations; electronic supplementary material, table S1). We then extracted 12 acoustic features from vocalizations for the individual distinctiveness analysis (table 2), using a custom script [42–45] in Praat software [46]. Eight of these features were also extracted for the vocal repertoire analysis (i.e. all features except those related to the fundamental frequency, which were not available for soft snorts that are not tonal). Additionally, to explore the vocal repertoire, mel-spectrograms were generated from audio files using STFT, following Thomas *et al*. [28]. Spectrograms were padded with zeros according to the length of the longest audio file to ensure uniform length for all files, and time-shift adjustments were implemented to align the starting points of vocalizations [28].

**Table 2.** Vocal features extracted from zebra vocalizations.

feature	description
mean *F*0 (Hz)	mean value of the fundamental frequency, i.e. lowest frequency of the sound
max *F*0 (Hz)	maximum value of the fundamental frequency
min *F*0 (Hz)	minimum value of the fundamental frequency
range *F*0 (Hz)	max *F*0–min *F*0
*Q*25% (Hz)	frequency below which 25% of the energy is contained
*Q*50% (Hz)	frequency below which 50% of the energy is contained
*Q*75% (Hz)	frequency below which 75% of the energy is contained
peak frequency (Hz)	frequency of maximum amplitude
duration (s)	total duration of a vocalization
amplitude variation (dB s^−1^)	cumulative variation in amplitude divided by the total vocalization duration
amplitude modulation rate (s^−1^)	ratio of complete amplitude cycles to the total duration of the vocalization
amplitude modulation extent (dB)	mean peak-to-peak variation of each amplitude modulation

### 2.4. Statistical analyses

#### 2.4.1. Vocal repertoire

We applied both supervised and unsupervised machine learning to both acoustic features and spectrograms using Python (v. 3.9.7) [47].

##### 2.4.1.1. Supervised method

To define the vocal repertoire through an acoustic feature-based approach, we deployed feature importance analysis by SHapley Additive exPlanation (SHAP) [48], using the *shap* library (v. 0.40.0) [49]. Six features with SHAP value >1 were selected (electronic supplementary material, figure S1). We split the selected features with vocalization type labels into a training dataset (70%) and a testing dataset (30%) using the *Scikit-learn* library (function: train_test_split, v. 0.24.2) [50]. Subsequently, we employed a supervised approach, the eXtreme Gradient Boosting (XGBoost) classifier in *xgboost* library (v. 1.6.0) [51] to train the model. Three hyperparameters were tuned on the training dataset to reach maximum accuracy using *optuna* library (direction = minimize, n_trials = 200, v. 2.10.0) [52], incorporating cross-validation (five folds), which resulted in the best model (electronic supplementary material, table S5).

To define the vocal repertoire through a spectrogram-based approach, we split the dataset into a training set (49%), a validation set (21%) and a test set (30%), using the *Scikit-learn* library (function: train_test_split, v. 0.24.2) [50]. We implemented a CNN architecture using the *tensorflow* library (v. 2.8.0) [53]. The architecture was constructed (electronic supplementary material, table S6) and seven hyperparameters were tuned to reach maximum accuracy on the training and validation dataset using the *optuna* library (direction = minimize, n_trials = 50, v. 2.10.0) [52], which resulted in the best model (electronic supplementary material, table S6).

We evaluated model performance for both feature-based and spectrogram-based classification models through predictions on each test dataset, including the test accuracy across all call types (number of correct predictions/total number of predictions), and three metrics for each call type: precision (true positives/(true positives + false positives)), recall (true positives/(true positives + false negatives)) and the harmonic mean of precision and recall—*f*1-score (2 × (precision × recall)/(precision + recall)) [54]. We also plotted the confusion matrix between true classes and predicted classes.

##### 2.4.1.2. Unsupervised method

For both acoustic feature-based and spectrogram-based analyses, we applied uniform manifold approximation and projection (UMAP) in the *umap* library (function: umap.UMAP, n_neighbors = 200 and local_connectivity = 150 for acoustic feature-based analysis and metric = calc_timeshift_pad and min_dist = 0 for spectrogram-based analysis, v. 0.1.1) [55], to reduce variables into a two-dimensional (2D) latent space. We also implemented *k*-means clustering algorithm for both analyses from the *Scikit-learn* library (function: kmeans.fit, v. 0.24.2) [50], to identify distinct clusters using the elbow method [56]. The acoustic feature-based analysis followed the same feature importance selection result as in the feature-based supervised method (six features), while the spectrogram was analysed using scripts provided by Thomas *et al*. [28]. We drew the 2D latent space and clusters using *matplotlib* library (v. 3.4.3) [57]. We also plotted the confusion matrix between true classes and predicted clusters using the *seaborn* library (v. 0.11.2) [58]. Finally, we plotted the pairwise distances within a vocalization type against between vocalization types using the script provided by Thomas *et al*. [28].

### 2.4.2. Vocal Individuality

We assessed the individual distinctiveness of vocalization types using R studio (v. 2022.02.1 with R v. 4.2.2) [59,60].

We performed a Kaiser–Meyer–Olkin test on the 12 acoustic features to measure the suitability of those features for factor analysis, using the *psych* package (KMO function, v. 2.4.2 [61]). Variables with measure of sampling adequacy (MSA) equal to or greater than 0.5 (electronic supplementary material, table S7) [62] were selected, and subsequently input into a principal component analysis (PCA) using the *stats* package (prcomp function, v. 4.2.2), to reduce correlation and multicollinearity [63]. PC loadings with eigenvalues >1 (electronic supplementary material, table S9) were then first input into a discriminant function analysis (DFA) with individual identity as the grouping factor, using the MASS package (Ida function, v. 7.3–58.2) [64], to visualize the feature (PC) loadings responsible for individuality. They were then additionally input into a permuted discriminant function analysis (pDFA), to assess individual distinctiveness using functions developed by Mundry & Sommer [65], which are based on the MASS package [64]. We ran a first nested pDFA with sex as a restriction factor, and a second nested pDFA with location as a restriction factor [65]. Both pDFAs included individual identity as the test factor.

## 3. Results

### 3.1. Vocal repertoire

**Figure 2.**
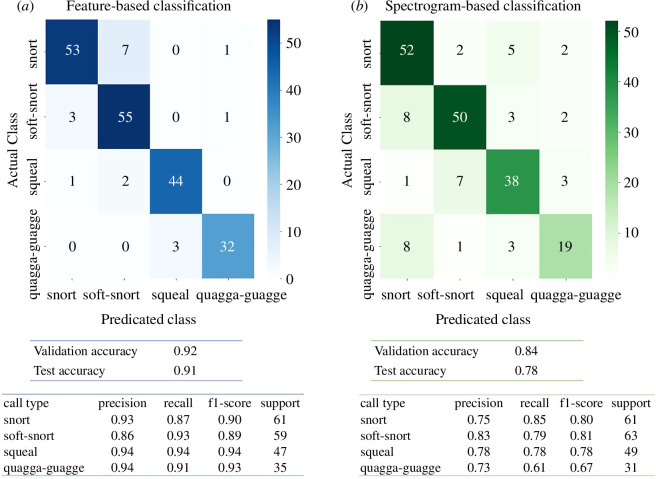
Supervised classification results for (*a*) feature-based analysis and (*b*) spectrogram-based analysis on the test dataset, shown by the confusion matrix (actual class versus predicted class). Overall validation and metrics (electronic supplementary material, table S7) evaluating the model performance are also indicated.

The feature-based supervised classification achieved a 91% test accuracy across the four vocalization types identified manually during labelling. The quagga quagga and the squeal revealed the highest *f*1-score at 93% and 94%, respectively, followed by the soft snort (89%) and snort (90%) (figure 2*a*).

In comparison, the spectrogram-based supervised classification yielded a lower test accuracy of 78%. The quagga quagga had the lowest *f*1-score at 67%, while the snort, soft snort and squeal had relatively higher *f*1-scores at 80%, 81% and 78%, respectively (figure 2*b*). Misclassifications primarily involved quagga quagga and soft snorts being categorized as snorts, while most misclassified squeals were classified as soft snorts (figure 2*b*).

**Figure 3.**
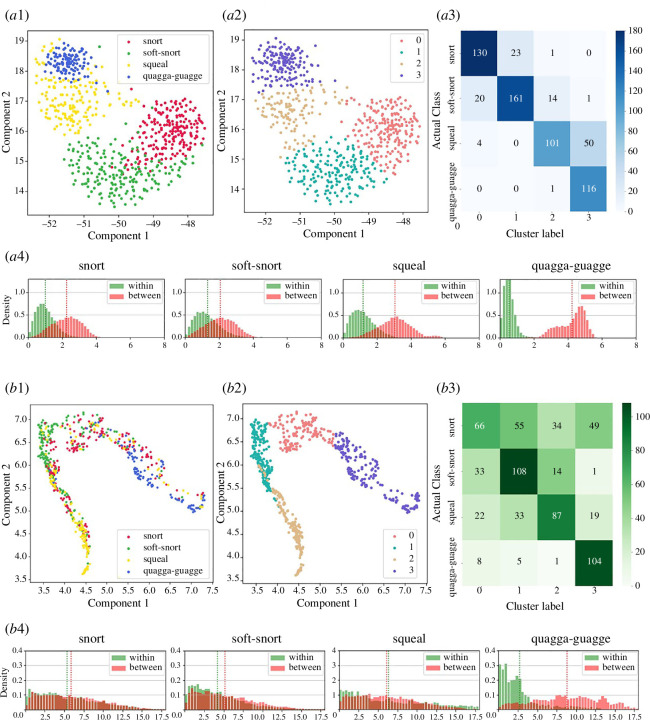
Unsupervised clustering result for (*a*) feature-based analysis and (*b*) spectrogram-based analysis on full dataset: (*a*1,*b*1) dimensionality reduction result from UMAP algorithm; (*a*2,*b*2) clustering result through *k*-means; (*a*3,*b*3) confusion matrix of actual vocalization types against clusters; (*a*4,*b*4) overall distances within a vocalization type (‘within’—green) versus between vocalizations of different types (‘between’—red), where the more separated the two distributions, the more distinct the cluster.

Feature-based unsupervised clustering resulted in four clusters based on the elbow method, with a clear separation between tonal vocalizations (the quagga quagga and the squeal) and non-tonal ones (the snort and the soft snort) (figure 3*a*1,*a*2). The quagga quagga had the most distinct cluster among all types, with 99% of vocalizations classifying into one cluster (figure 3*a*3), along with the most obvious separations of within–between density distributions (figure 3*a*4). The squeal showed a clear separation of within–between density distributions (figure 3*a*4), but only 65% of squeals fell into one cluster, while the others were clustered as quagga quagga (figure 3*a*3). The snort and the soft snort showed less clear separations of within–between density compared with the other two vocalization types, while reaching a high proportion of vocalizations categorizing as one cluster (88% for the snort and 82% for the soft snort).

Spectrogram-based unsupervised clustering displayed four clusters based on the elbow method (figure 3*b*1,*b*2), which revealed the clearest cluster for quagga quagga (89% quagga quagga were classified into one cluster), while the other three types showed less clarity (figure 3*b*1,*b*3,*b*4). The squeal and soft snort exhibited a moderate level of clustering, with more than half of the calls falling into one cluster (soft snorts: 55%, squeals: 54%; figure 3*b*1,*b*3), and strong overlapped within–between density distributions for both types (figure 3*b*4). The snort displayed the least clear cluster, distributed evenly across four clusters (17–32%; figure 3*b*1,*b*3,*b*4).

Combining results from supervised and unsupervised machine learning algorithms across vocal features and spectrograms, our findings suggest that plains zebras exhibit at least four distinct vocalization types: snorts, soft snorts, squeals, and quagga quagga.

### 3.2. Vocal individuality

Snorts were classified to the correct individual (*n* = 18) significantly above chance level when controlling for both sex (correctly cross-classified percentage, chance level: 13.32%, 4.69%) and location (13.56%, 6.28%; *p* = 0.001 for both; table 3). In contrast, among 12 individuals, the percentage of correctly cross-classified squeals was significantly above chance level only when controlling for sex (correctly cross-classified percentage, chance level: 15.02%, 7.47%, *p* = 0.022), while location-controlled results did not differ from chance (14.08%, 11.06%; *p* = 0.216; table 3).

**Table 3.** Results of the pDFA applied to snorts and squeals, controlled by either sex or location (*p* < 0.05 shown in bold). Number of correctly cross-classified vocalizations, expected number of correct cross-classified vocalizations, percentage of correctly cross-classified vocalizations and corresponding cross-classified chance level and statistical significance (*p*-value).

	snort			squeal	
	control (sex)	control (location)		control (sex)	control (location)
no. of correct cross-classified vocalizations	29.16	29.70		10.21	9.58
expected no. of correct cross-classified vocalizations	10.26	13.76		5.08	7.52
correctly cross-classified percentage	13.32%	13.56%		15.02%	14.08%
cross-classified chance level	4.69%	6.28%		7.47%	11.06%
*p*-value for cross-classified	**0.001**	**0.001**		**0.022**	0.216

For snorts, DF1 and DF2 accounted for 88.02% of the variance (electronic supplementary material, table S8). DF1 was highly correlated (|*r*|≥0.5) with scores from PC1 and PC2 (electronic supplementary material, table S8), which represented *F*0-related features, energy distribution (*Q*25%, *Q*50% and *Q*75%), as well as duration and amplitude modulation (AM)-related features (electronic supplementary material, table S9). DF2 had a strong correlation (|*r*| ≥ 0.5) with scores from PC1 and PC3 (electronic supplementary material, table S8), which were correlated with *F*0-related features, duration and AM-related features (electronic supplementary material, table S9).

For squeals, DF1 and DF2 contributed 68.72% of the variance (electronic supplementary material, table S8). DF1 was strongly correlated (|*r*| ≥ 0.5) with PC2 scores (electronic supplementary material, table S8), which included *F*0-related features, duration and amplitude variation (electronic supplementary material, table S9). DF2 was strongly correlated (|*r*| ≥ 0.5) with scores from PC3 and PC4 (electronic supplementary material, table S8), which represented peak frequency, *Q*75% and AM-related features (electronic supplementary material, table S9).

Overall, our result suggests that plains zebra snorts, and to a lesser extent squeals, contain information about individual identity.

## 4. Discussion

The plains zebra is renowned for its loud and specific vocalizations, but investigations into its vocal repertoire and individuality have been limited. We employed feature-based and spectrogram-based machine learning for supervised classifications and unsupervised clustering to identify distinct vocalization types. Our findings revealed at least four vocalization types: the ‘snort’, the ‘soft snort’, the ‘squeal’ and the ‘quagga quagga’. We also analysed the vocal distinctiveness of two identified vocalization types, and found that snorts displayed significant differences between individuals, while squeals showed comparatively less individuality. This study uses state-of-the-art tools to estimate the repertoire size of this species, and hence could inspire future comprehensive explorations in a wider range of taxa and animal communication systems.

In order to reduce subjective biases, we investigated vocalization types present in plains zebra repertoire using both supervised and unsupervised machine learning algorithms. Our study improves the robustness of identifying distinct vocalization types compared with prior subjective descriptions [29,32]. Three of the four vocalization types that we found align with previous literature [29,32]: the ‘quagga quagga’ (corresponding to the previously described ‘bark’, or ‘i-ha’, used as a contact call for long-distance communication), the ‘snort’ (previously described as the ‘loud snort’, produced when moving into potentially dangerous cover, and the ‘long drawn-out snort’, the ‘whuffle’, the ‘blow’ or the ‘long snort’, emitted in contexts previously described as ‘contentment’) and the ‘squeal’ (previously described as the ‘chirp’, appearing during aggression or conflict, but also greeting and play). The ‘snort’ merges the previously described ‘short snort’ and ‘long snort’ [29,32]. Classification from both acoustic features and spectrogram, together with clustering results for acoustic features, supported this conclusion. However, spectrogram clustering may imply potential subdivisions within the ‘snort’. Notably, the ‘long drawn-out wail’ (previously recorded when a foal is in distress) and the ‘two-syllable alarm’ (previously described as emitted when zebras sight predators) mentioned in one of the previous studies [29] were not identified in our study. Further research across zebra subspecies is recommended for a comprehensive understanding of their vocal repertoire. Moreover, our data did not contain samples of all vocalization types at all locations, so we could not include location as a factor in the analyses of the vocal repertoire. We recommend that future studies examine this effect using a more balanced dataset.

The overall accuracy of both supervised and unsupervised classification analyses was much higher for the acoustic feature-based analysis than the spectrogram-based one. This difference between analysis types can be explained by the representativeness of the extracted acoustic features, which describe key attributes of each vocalization type, while spectrograms may capture excessive details that are not relevant for distinguishing vocalization types, or alternatively too insufficient details. This is supported by the fact that, in CNN analysis, local features are more important than global features [66]. Nevertheless, regarding specific vocalization types, divergent outcomes emerged from distinct analyses. For example, the acoustic feature-based classifier yielded higher *f*1-scores for tonal vocalizations (93% for the quagga quagga and 94% for the squeal) compared with non-tonal ones (90% for the snort and 89% for the soft snort), while the spectrogram-based classifier resulted in a much lower *f*1-score for the quagga quagga (67%) compared with the other three types (80% for the snort, 81% for the soft snort and 78% for the squeal). As another example, in clustering analyses, the acoustic feature-based analysis showed that squeals displayed the least clear cluster among the four types, while the spectrogram-based analysis revealed that snorts had the least clear cluster.

Our results revealed that the acoustic structure of snorts displays significant differences between individuals, when controlling for variation linked to both sex and location. This finding aligns with a similar study on southern white rhinoceros (*C. simum simum*), where snorts also showed individual distinctiveness, although less than other vocalization types [35]. Zebras emit snorts during various context (i.e. grazing and moving), suggesting the potential use of snorts to convey individual information and recognize group members. In addition, we would recommended future studies to investigate other factors influencing vocalizations, such as sex, age and emotions [67].

Our findings for squeals showed that this vocalization type displayed significant individual differences only when controlling for sex, but not for location. Squeals are primarily emitted during close social interactions, where visual or tactical signals are available. This may contribute to their limited individual distinctiveness, as zebras may use alternative modalities for individual recognition in this context. However, sex and location were confounded (two males from PNP, five and seven females from GKZ and KSP, respectively), preventing us from adequately controlling for one factor independently of the other. This imbalance in the data should thus be taken into consideration. Overall, the higher individuality in snorts compared with squeals supports the ‘distance communication hypothesis’, as snorts, being louder (B.X. 2021, personal observation) with a lower fundamental frequency (electronic supplementary material, table S1), probably propagate over longer distances than squeals that are rather quiet in plains zebras, thus conveying more information on individuality owing to the lack of available visual cues over long distances [68].

In conclusion, our exploration of the vocal repertoire of plains zebras suggests at least four distinct vocalization types: the ‘snort’, the ‘soft snort’, the ‘squeal’ and the ‘quagga quagga’. We also found that snorts are more individually distinct than squeals. We recommend the combined use of supervised and unsupervised learning, on both acoustic features and spectrogram in future studies investigating vocal repertoires. We also recommend further explorations into the vocal repertoire of zebras across subspecies, and investigations of the individual distinctiveness of more vocalization types (e.g. quagga quagga and soft snort).

## Data Availability

The datasets, audio recordings and scripts used in this study are available at Dryad [45]. Supplementary audio, tables and figures are also available at Dryad.
